# Risk factors and outcomes associated with difficult-to-treat resistance in *Stenotrophomonas maltophilia*: A clinical and microbiological hospital-based cohort study

**DOI:** 10.1017/S0950268826101708

**Published:** 2026-06-11

**Authors:** Patricio Ross, Kasim Allel, Vicente Gándara, Gonzalo Peralta, Francisca Caro, Claudia Castillo, Javiera Jiménez, Jorge Miles, Rodolfo Amstein, Patricia García, Jaime Labarca

**Affiliations:** 1 Departamento de Enfermedades Infecciosas del Adulto, Pontificia Universidad Catolica de Chile, Chile; 2 Enfermedades Infecciosas del Adulto, Red de Salud UC Christus, Chile; 3 Nuffield Department of Primary Care Health Sciences, University of Oxford, UK; 4 Subdirección de Gestión Asistencial, Servicio de Salud Arica y Parinacota, Chile; 5 Laboratorio de Microbiología, Red de Salud UC Christus, Chile; 6 Departamento de Laboratorios Clínicos, Pontificia Universidad Catolica de Chile, Chile; 7 Departamento de Medicina Interna, Pontificia Universidad Catolica de Chile, Chile; 8 Departamento de Pediatría, Pontificia Universidad Catolica de Chile, Chile

**Keywords:** antimicrobial resistance, antimicrobial stewardship, hospital-acquired infection, metallo-*β*-lactamase, *Stenotrophomonas maltophilia*

## Abstract

We aimed to comprehensively assess *Stenotrophomonas maltophilia* infections, examining their incidence and clinical and microbiological characteristics in a tertiary hospital between 2021 and 2023. Primary outcomes were 30-day mortality and desirability-of-outcome ranking (DOOR). *S. maltophilia* incidence rose from 0.38 in 2017 to 2.66 cases/1000 patient-days in 2023, correlated with carbapenem consumption (rho = 0.90, *p* = 0.04). The cohort included 101 patients (median age of 61 years, IQR 43–71); 63% were male patients, 91% admitted in ICU, predominantly with lower respiratory tract infections (75%). Difficult-to-treat resistance (DTR) was identified in 14% of the isolates and associated with previous trimethoprim–sulfamethoxazole (TMP–SMX) use (aOR 23.15, 95% CI 3.67–145.77). Thirty-day mortality was 25% and associated with invasive mechanical ventilation (aOR 5.80, 95% CI 1.03–32.63), while appropriate definitive therapy was protective (aOR 0.01, 95% CI <0.01–0.16). Worse DOOR outcomes were associated with levofloxacin-resistant isolates (69.3%, CI 55.9%–80.9%) and inappropriate definitive treatment (81.1%, 95% CI 68.2%–90.8%). Overall, *S. maltophilia* has emerged as a relevant carbapenem-resistant gram-negative bacteria, with a high frequency of DTR phenotypes associated with previous TMP–SMX exposure. Appropriate therapy and levofloxacin susceptibility were linked to better outcomes.

## Introduction

*Stenotrophomonas maltophilia* is an emergent, opportunistic gram-negative bacillus that causes lower respiratory tract infections and bloodstream infections in critical and immunosuppressed patients [1–4]. It has a wide array of resistance mechanisms, including intrinsic inducible metallo-*β*-lactamases (*bla*_L1_), rendering most *β*-lactams ineffective, including carbapenems [1]. Current guidance suggests trimethoprim–sulfamethoxazole (TMP–SMX) and levofloxacin as first-line agents, preferably in combination [5].

Historically, antimicrobial resistance to first-line agents in *S. maltophilia* has been low [6, 7]. In the SENTRY antimicrobial surveillance programme, which analysed over 6000 isolates between 1999 and 2016, resistance rates were 19.5% for levofloxacin and 4.4% for TMP–SMX resistance, respectively. Notably, the frequency of resistance to both agents (i.e. difficult-to-treat resistance or DTR) was only 1.7%, with Chile reporting one of the highest rates at 5.8% [6]. A recent update, including isolates from 2018 to 2023, revealed a similar global DTR rate of 2.1% [8].

Since the COVID-19 pandemic, our centre has observed a rise in *S. maltophilia* infections and increasing resistance rates. We conducted a retrospective cohort study to (i) describe temporal trends in *S. maltophilia* infections; (ii) quantify the frequency of and identify risk factors associated with DTR; and (iii) evaluate factors associated with clinical outcomes, including 30-day mortality.

## Methods

### Study design

We performed a retrospective cohort study of hospitalized patients with *S. maltophilia* infection between January 2021 and December 2023 in the Hospital Clínico de la Universidad Católica (HCUC), a 450-bed referral hospital in Santiago de Chile, belonging to the Red de Salud UC-CHRISTUS health network. Eligible patients were assessed from laboratory data. The study population consisted of adult hospitalized patients, including both critically ill patients admitted to the ICU and patients in medical and surgical wards in a tertiary-care setting. Patients were included if (i) they were adults, including ICUs, medical and surgical ward admissions and (ii) they presented clinical infections following cultures and physician criteria. Patients were excluded when (i) colonization only was considered; (ii) there was no intention to treat due to short life expectancy, defined as a documented decision by the treating team to prioritize comfort or end-of-life care; and (iii) follow-up was not conducted due to hospital transfer or outpatient treatment. This study is reported according to the STROBE guidelines; the checklist is provided in Supplementary Table A1.

### Clinical, microbiological, and treatment characterization

We evaluated age, sex, comorbidities, immunosuppression (transplant, cancer, or pharmacological), among others. Appropriate empirical antimicrobial therapy was defined as TMP–SMX, levofloxacin, minocycline, or ceftazidime/avibactam plus aztreonam (CZA–ATM) initiated within 48 h after the index culture provided the isolate as susceptible. Appropriate definitive therapy was defined according to in-vitro susceptibility. CZA–ATM was considered as monotherapy due to the inactivity of both agents by themselves.

Isolates were identified via matrix-assisted laser desorption/ionization (MALDI, Bruker®) and tested for TMP–SMX (agar diffusion), levofloxacin and minocycline (disk diffusion or E-test), and CZA–ATM (synergy testing with overlaid strips) [9]. MIC_50_/_90_ was determined for CZA–ATM in the absence of Clinical and Laboratory Standards Institute (CLSI) cutoffs. Predefined susceptibility patterns were categorized as follows: multidrug-susceptible (MS) isolates (i.e. susceptible to both TMP–SMX and levofloxacin); isolates resistant only to TMP–SMX; isolates resistant only to levofloxacin; and difficult-to-treat resistant (DTR) isolates, defined as resistance to both agents.

High-incidence period isolates were analysed for clonality using pulsed-field gel electrophoresis (PFGE) with SpeI restriction enzyme, interpreted according to Tenover’s criteria [10]. We used GelJ® software to generate a dendrogram for clonal analysis.

### Outcomes

Our primary outcome was 30-day all-cause mortality since the index culture. We also computed a five-level Desirability of Observed Outcome Ranking (DOOR) [11, 12], ranging from 0 (alive without deleterious events) to 4 (death). Intermediate DOOR categories (levels 1–3) were assigned according to the cumulative number of deleterious events, with each predefined event contributing 1 point. Events included lack of clinical response, failed discharge, or adverse events (e.g. acute kidney failure or infection by *C. difficile*). More information is detailed in the Supplementary material (Supplementary Table A2). Secondary outcomes included microbiological eradication, total length of stay (LOS, in days), recurrence (defined as new symptoms with positive cultures to *S. maltophilia* after treatment completion), and hospital readmission. Pre-specified subgroups included immunosuppressed patients, COVID-19 diagnosis as the reason for hospital admission, recent major surgery, presence of bacteraemia, resistance pattern, and antimicrobial treatment.

### Incidence density

Following recommendations for MDRO surveillance from SHEA/HICPAC and the Chilean Collaborative Group for Bacterial Resistance [13, 14], ICU incidence was calculated as incidence density, expressed as cases per 1000 patient-days, by dividing the number of first positive *S. maltophilia* cultures by the occupation of our 32-bed ICU expressed in patients-day (January 2017–December 2023). Carbapenem consumption was calculated as defined daily doses (DDDs) per 1000 patient-days. For context, we compared the incidence in the same units for carbapenem-resistant *Pseudomonas aeruginosa* (CR-PA), *Acinetobacter baumannii* (CRAB), and Enterobacterales (CRE), including Carbapenemase-producing Enterobacterales (CPE).

### Statistical analysis

For the clinical cohort, no sample size was prespecified as consecutive sampling was performed for the period of interest. Descriptive statistics included medians with interquartile ranges for continuous variables, while frequencies and percentages were used for categorical variables. Analytical statistics consisted of T-tests or Mann–Whitney *U* tests for parametric and nonparametric variables, respectively. Chi-squared or Fisher’s exact tests were used for categorical variables. Multivariable logistic regression was performed to assess for confounding in variables associated with 30-day in-hospital mortality and DTR, both analysed as binary outcomes. Variables with *p* < 0.1 in univariable analyses were included, and only baseline covariates were considered as risk factors for DTR; odds ratios (ORs) with 95% confidence intervals (CI) were reported. Furthermore, we calculated the probability that a randomly selected patient with a resistant strain (levofloxacin, TMP–SMX, and DTR) versus a susceptible respective strain had a less desirable DOOR scores using pairwise analyses. A probability of 50% indicated no difference between the DOOR distributions of the two groups. A probability >50%, with a 95% confidence interval (CI) excluding 50%, indicated inferiority of the resistant strain compared to the susceptible strain. Confidence intervals were determined using 10000 bootstrap resamples. Additionally, we assessed the difference in DOOR values and the 95% CI for an appropriate definitive therapy, compared to inadequate. Robust standard errors were used in regression analyses. To assess the impact of carbapenem consumption, the incidence of *S. maltophilia* infections was correlated with carbapenem consumption using Spearman’s rank correlation coefficient (rho), given the non-parametric distribution of the variables. We did not present missing observations. All statistical analyses were performed in Stata 18.

### Ethics approval

This study was approved by the institutional review board (Clinical Ethics Committee from Pontificia Universidad Católica de Chile, ID 221207007). Informed consent was waived due to the retrospective design.

## Results

### Cohort characteristics

A total of 584 patients with positive cultures were screened. After removing duplicates and applying the inclusion and exclusion criteria, we enrolled 101 unique patients (Supplementary Figure A2). Median age was 61 (IQR 43–71), 63.4% were male patients, and the median age-adjusted Charlson Comorbidity Index was 3 (IQR 2–6). Most patients were critically ill, with 92 (91.1%) admitted to the ICU at the time of diagnosis (Table 1). The main sources of infection were lower respiratory tract infection (LRTI, 75.2%) and primary bloodstream infection (14 patients, 13.8%). A total of 19 (19%) of patients were bacteraemic. Polymicrobial cultures were present in 57 (56.4%) patients, and the most frequent co-isolated pathogen was *P. aeruginosa* (22 patients, 21.8%). Regarding patient profile, immunocompetent COVID-19 patients predominated early in 2021, while immunosuppressed non-COVID-19 patients were most affected in 2023. Prior antibiotic exposure was highly prevalent (87.1%), whereas prior carbapenem use was less frequent (45.5%), with no significant different across study years, suggesting that *S. maltophilia* infections occurred in the context of broad antimicrobial exposure, but not exclusively carbapenems. (Supplementary Table A3). Patients with DTR were more frequently immunosuppressed, had prior *S. maltophilia* colonization, and were more likely to have received prior antibiotic exposure, particularly TMP–SMX (Table 1).

**Table 1.** Clinical characteristics of patients with *S. maltophilia* infection according to antimicrobial resistance pattern (*N* = 101) Table 1. long description.

Characteristics/subgroups	MS (count, %) 69 (68.3%)	Resistance to TMP–SMX (count, %) 12 (11.9%)	Resistance to LVX (count, %) 6 (5.9%)	DTR (count, %) 14 (13.9%)	T- or χ^2^-test^↑^ *p*-value
Age (in years)	59 (44; 68)	72 (50; 76)	70 (36; 74)	64 (39; 74)	0.893
Male sex (count, %)	47 (68.1%)	7 (58.3%)	2 (33.3%)	8 (57.1%)	0.339
Obesity (count, %)	49 (71.0%)	10 (83.3%)	6 (100.0%)	13 (92.9%)	0.140
Previous hospital admission (count, %)	17 (24.6%)	2 (16.7%)	2 (33.3%)	5 (35.7%)	0.689
Charlson Comorbidity Index (CCI) (mean, CIs)	3 (2; 5)	5 (2; 8)	5 (2; 6)	4 (3; 7)	**0.023**
COVID-19 as reason for admission (count, %)	43 (62.3%)	9 (75.0%)	5 (83.3%)	11 (78.6%)	0.455
Immunosuppression (count, %)	15 (21.7%)	1 (8.3%)	1 (16.7%)	8 (57.1%)	**0.017**
Surgery during admission (count, %)	24 (34.8%)	3 (25.0%)	1 (16.7%)	6 (42.9%)	0.626
*S. maltophilia* colonization (count, %)	4 (5.8%)	1 (8.3%)	2 (33.3%)	4 (28.6%)	**0.023**
Intensive care unit admission (count, %)	63 (91.3%)	11 (91.7%)	6 (100.0%)	12 (85.7%)	0.778
Previous antibiotic use (count, %)	64 (92.8%)	8 (66.7%)	3 (50.0%)	13 (92.9%)	**0.003**
TMP–SMX (count, %)	7 (10.1%)	0 (0.0%)	1 (16.7%)	10 (71.4%)	**<0.001**
Levofloxacin (count, %)	5 (7.2%)	1 (8.3%)	0 (0.0%)	2 (14.3%)	0.720
Ceftriaxone (count, %)	29 (42.0%)	4 (33.3%)	1 (16.7%)	5 (35.7%)	0.626
Piperacillin/Tazobactam (count, %)	37 (53.6%)	5 (41.7%)	1 (16.7%)	9 (64.3%)	0.220
Carbapenems (count, %)	31 (44.9%)	4 (33.3%)	3 (50.0%)	8 (57.1%)	0.673
Central venous catheter (count, %)	46 (66.7%)	6 (50.0%)	3 (50.0%)	9 (64.3%)	0.634
Invasive mechanical ventilation (count, %)	48 (69.6%)	5 (41.7%)	3 (50.0%)	7 (50.0%)	0.170
Renal replacement therapy (count, %)	13 (18.8%)	1 (8.3%)	0 (0.0%)	3 (21.4%)	0.523
SOFA Score (mean, CIs)	6 (3; 9)	2 (1; 6)	6 (4; 9)	5 (3; 8)	0.250
APACHE II Score (mean, CIs)	17 (12; 22)	9 (7; 18)	16 (14; 19)	16 (12; 24)	0.220

*Notes:* Categorical variables expressed as frequency and percentages. Continuous variables expressed as median and interquartile ranges. DTR: Difficult-to-treat resistance. LRTI: Lower respiratory tract infection. LVX: Levofloxacin. MS: Susceptible to levofloxacin and trimethoprim-sulfamethoxazole. TMP–SMX: Trimethoprim–sulfamethoxazole. aOR: Adjusted Odds Ratios. CI: Confidence intervals. ☨ T- or χ^2^-tests were performed following variable’s distribution via comparing mean differences across all subgroups.

### Microbiological characterization

**Figure 1.**
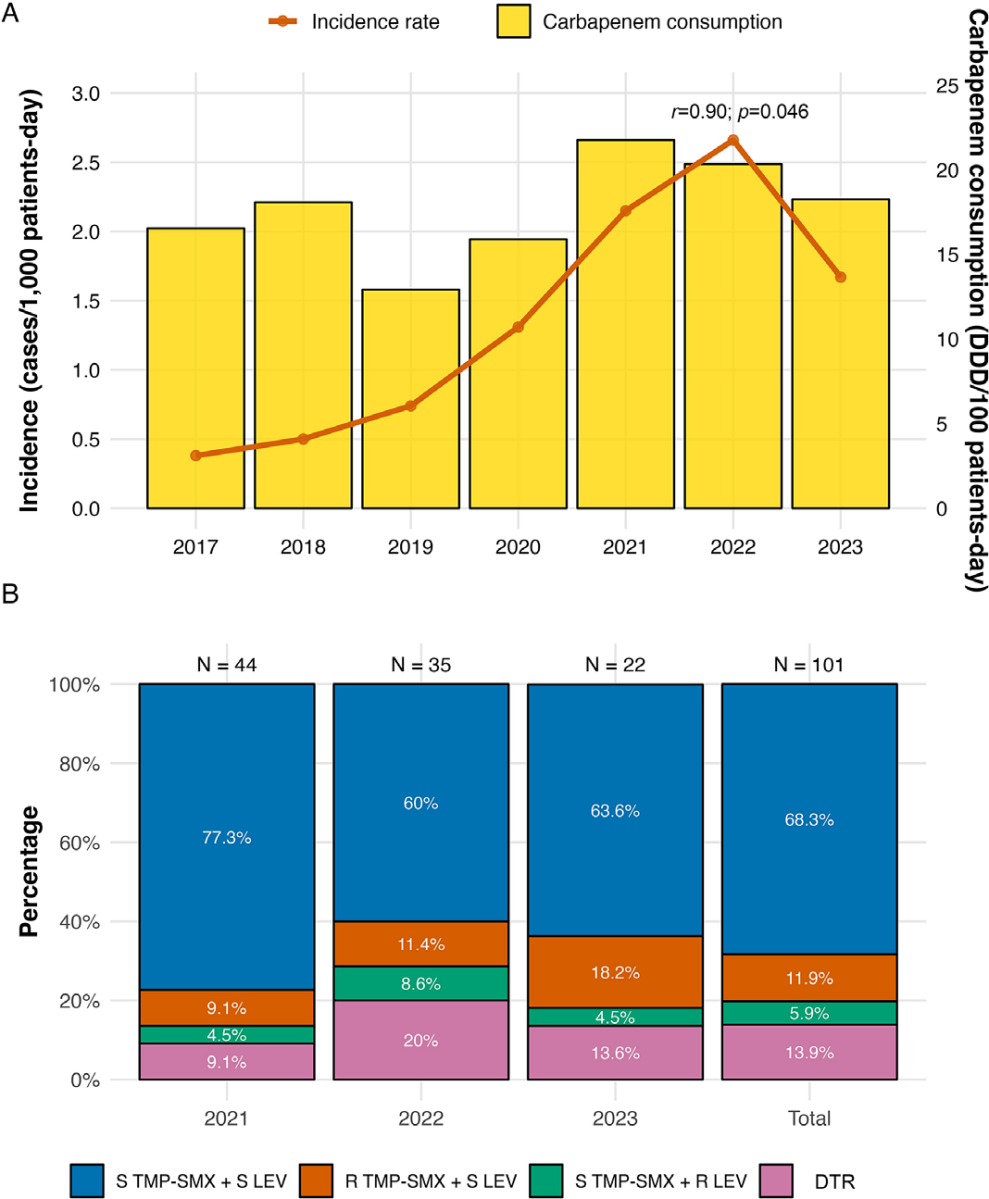
(a) *Stenotrophomonas maltophilia* incidence rate and carbapenem consumption in ICU and (b) Antimicrobial susceptibility profiles in the hospital. *Notes:* Carbapenems included ertapenem, imipenem, and meropenem. DDDs: Defined daily doses; TMP–SMX: Trimethoprim/sulfamethoxazole; LEV: Levofloxacin; S: Susceptible; R: Resistant. Figure 1. long description.

**Figure 2.**
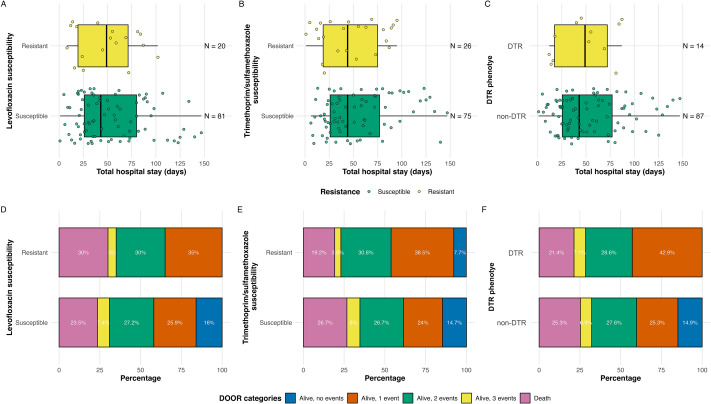
Clonal analysis of *S. maltophilia* isolates obtained during the high-incidence period in the intensive care unit. *Notes:* PT: Pulsotypes. (A) Pulsed-field gel electrophoresis (PFGE) patterns and dendrogram of analysed isolates. (B) Distribution of pulsotypes among isolates. Pulsotypes were arbitrarily numbered. Number of isolates for each pulsotype are presented. Figure 2. long description.

**Figure 3.**
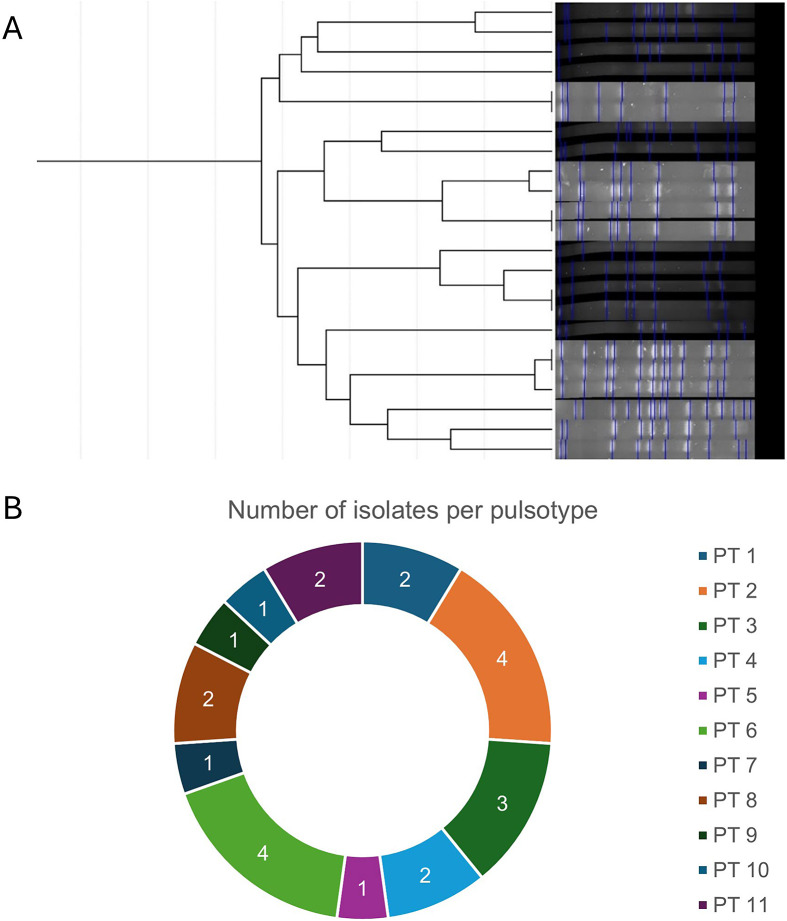
Length of hospital stay and desirability of outcome ranking (DOOR) of patients with *S. maltophilia* infection, presented by resistance type. *Notes:* DOOR: Desirability outcome raking; DTR: Difficult-to-treat resistance, considering levofloxacin and trimethoprim/sulfamethoxazole. Figure 3. long description.

Resistance to TMP–SMX and levofloxacin was present in 26 (25.7%) and 20 (19.8%) isolates, respectively, while 14 (13.9%) isolates were DTR (Table 1, Figure 1b). All isolates were susceptible to minocycline. Susceptibility to CZA–ATM was tested in 79 isolates, with an MIC_50/90_ of 1/2 mg/L, respectively. In univariate analysis, the DTR phenotype was associated with a higher Charlson Comorbidity Index, previous TMP–SMX use, immunosuppression, and bacteraemia. Multivariate analysis confirmed the association between previous TMP–SMX use (aOR 23.15, 95% CI 3.67–145.77) and the Charlson Comorbidity Index (aOR 1.44, 95% CI 1.04–2.00) (Table 2). Twenty-three isolates were selected from patients whose infection occurred within 30 days of each other and within the same hospital unit for clonality assessment. PFGE identified 11 pulsotypes (Figure 2, 3) of which included 11 isolates.

**Table 2.** Multivariable analysis of risk factors associated with difficult-to-treat resistance (DTR) in patients with *S. maltophilia* infection Table 2. long description.

Characteristics	Non-DTR STMA *n* = 87 (86.1%)	DTR STMA *n* = 14 (13.9%)	*p*-value	aOR	CI 95%	*p*-value
Charlson comorbidity index	3 (2; 6)	4 (3; 7)	0.091	1.44	1.04–2.00	**0.025**
Previous TMP–SMX use	8 (9.2%)	10 (71.4%)	<0.001	23.15	3.67–145.77	**<0.001**
Immunosuppression	17 (19.5%)	8 (57.1%)	0.002	2.19	0.33–14.45	0.414
Bacteriaemia	13 (14.9%)	6 (42.9%)	0.013	5.51	0.74–40.92	0.095

*Notes:* Categorical variables expressed as frequency and percentages. Continuous variables expressed as median and interquartile ranges. TMP–SMX: Trimethoprim-sulfamethoxazole; DTR: Difficult-to-treat resistance; aOR: Adjusted Odds Ratios; CI: Confidence intervals; STMA: *S. maltophilia.*

### Antimicrobial treatment characteristics

Seventy-five (74.3%) patients received monotherapy, and 12 (11.9%) received combined therapy. Fourteen patients (13.9%) received ceftazidime, carbapenems, or aminoglycosides, not considered active agents. Fifty (49.5%) patients received active empirical treatment. Antibiotic treatment consisted mostly of TMP–SMX (39.6%) and levofloxacin-containing regimens (27.7%). Fifteen patients received minocycline, and another 15 received CZA–ATM. The median treatment duration was 10 (IQR 7–16) days.

### Health outcomes: 30-day in-hospital mortality and DOOR

Twenty-five (24.8%) of the 101 patients died 30 days from index culture. Thirteen of the 14 patients (93%) who did not receive appropriate treatment died after a median of 5 (1–9) days. On univariate analysis, patients who did not receive appropriate treatment had a higher mortality risk than those who did not (92.8% vs. 13.8%, *p* = <0.001), with no differences between prespecified subgroups (Table 3). No choice of antimicrobial agent was associated with lower mortality among those who received appropriate treatment; however, levofloxacin showed a non-significant trend towards lower mortality (aOR 0.12, 0.01–1.11) on multivariable analysis (Table 3). Logistic regression did show a significant association with mortality for invasive mechanical ventilation at diagnosis (aOR 5.80, 95% CI 1.03–32.63) and appropriate treatment (aOR 0.01, 95% CI <0.01–0.16).

**Table 3.** Risk factors for 30-day in-hospital mortality among patients infected with *S. maltophilia* (*N* = 101 patients) Table 3. long description.

Characteristics	Univariable (30-day mortality)			Multivariable (30-day mortality)		
	No (*n* = 76)	Yes (*n* = 25)	*p*-value	aOR	95% CI	*p*-value
**A. All patients (*n* = 101)**						
Age, years	60 (38–68)	67 (56–76)	0.094	1.02	0.98–1.06	0.195
Male	50 (65.8%)	14 (56.0%)	0.378	0.59	0.15–2.30	0.453
Invasive mechanical ventilation	43 (56.6%)	20 (80.0%)	0.036	6.10	1.07–34.81	**0.042**
APACHE II score	15 (9; 19)	22 (16; 25)	<0.001	1.06	0.96–1.18	0.195
Appropriate definitive treatment	75 (98.7%)	12 (48.0%)	<0.001	0.01	<0.01–0.17	**0.001**
**B. Only patients with appropriate definitive treatment (*n* = 87)**						
**Antibiotic therapy**						
Levofloxacin	27 (36.0%)	1 (8.3%)	0.057	0.12	0.01–1.11	0.063

*Notes:* Categorical variables expressed as frequency and percentages. Continuous variables expressed as median and interquartile ranges. TMP–SMX: Trimethoprim–sulfamethoxazole; aOR: Adjusted Odds Ratio; CI: Confidence interval.

The DOOR analyses showed that 13 (12.9%) patients were alive without events, 28 (27.7%) patients were alive with one event, 35 (34.6%) were alive with two or three events, and 25 (24.8%) were dead at 30 days from index culture, with no difference in univariate analysis between susceptibility patterns (Supplementary Table A4). In pairwise analyses, the probability of a random patient having an equal or worse DOOR result was 69.3% (95% CI: 55.9%, 80.9%) for levofloxacin-resistant isolates, 58.2% (95% CI: 45.8%, 69.9%) for TMP–SMX-resistant isolates, and 63.4% (95% CI: 50.9%, 76.3%) for DTR isolates, compared to their susceptible counterparts (Figure 3 D–F). Additionally, the probability of a random patient having a worse DOOR outcome was 81.1% (95% CI: 68.2%, 90.8%) if did not receive appropriate definitive antibiotic treatment.

Follow-up cultures were performed in 46 patients, achieving microbiological eradication in 27 (58.7%) of them. The median time from diagnosis to eradication was 4 days (4–19). Eradication was more frequent in bacteraemic against non-bacteraemic patients (12, 85.7% vs. 15, 46.9%, *p* = 0.01) and in those treated with levofloxacin versus those treated with other agents (11, 84.5% vs. 15, 48.4%, *p* = 0.026). Seventy-three patients (72.2%) were discharged alive, with a median of 29 days (18–60) from index culture. Infection recurred at 30 days in 13 (12.9%) patients, and only six patients were readmitted 30 days after discharge. Acute kidney injury and *C. difficile* infection occurred in 21 (20.8%) and 3 (3.0%) patients, respectively.

### Incidence

The overall incidence of *S. maltophilia* during the study period was 1.35 cases/1000 patient-days. *S. maltophilia* infection incidence rose from 0.38 cases/1000 patient-days in 2017 to 2.66 cases/1000 patient-days in 2022, representing a 7-fold increase, subsequently decreasing to 1.6 cases/1000 patient-days in 2023 (Figure 1A). CR-PA and CRE had higher and rising incidences, up to 5.2 and 7.2 cases/1000 patient-days, respectively. CPE had an initially lower, but equally rising incidence, with a maximum of 3.0 cases/1000 patient-days, surpassing *S. maltophilia* (Supplementary Figure A1). The incidence of CRAB was negligible during the whole period. The incidence of all CR-GNB peaked during 2022, with a following decrease of varying magnitude. Carbapenem consumption was correlated with *S. maltophilia* incidence (Spearman’s rho = 0.900, *p* = 0.046). No association between carbapenem consumption and incidence of CRE (rho = 0.700, *p* = 0.185), CR-PA (rho = 0.700, *p* = 0.239), CPE (rho = 0.700, *p* = 0.185) nor CRAB (rho = 0.6, *p* = 0.239) was observed.

## Discussion

*S. maltophilia* ICU incidence increased from 2017 to 2022, followed by a decline in 2023, and was correlated with carbapenem consumption. DTR was higher than previously reported and associated with prior TMP–SMX exposure. Appropriate definitive treatment was strongly associated with lower mortality, with levofloxacin showing a numerical survival advantage among treatment options. Levofloxacin resistance, conversely, was associated with worse DOOR outcomes.

Most patients were critically ill, with a high proportion requiring invasive mechanical ventilation. Risk profiles shifted from COVID-19 (2021) to immunosuppression (2023) as a main risk factor for infection. *S. maltophilia* has reemerged as an opportunistic bacterial secondary infection in COVID-19 patients [15, 16]. Consistent with previous reports, no difference in outcomes was observed in patients admitted for COVID-19 [17]. As expected, invasive devices were common; however, an unexpectedly low prior carbapenem use was found. Nevertheless, infection incidence was correlated with carbapenem consumption, supporting the notion that selective pressure by carbapenems may act from an ecological standpoint instead of an individual one.

TMP–SMX resistance was 26%, exceeding global (5–15%) and regional (5–10%) reports. The rate of levofloxacin resistance has been variable in literature (10–20%), with our cohort within that range (20%). Notably, our DTR rate (15%) is higher than previously reported (<2%) [6–8]. While risk factors for single-drug resistance have been reviewed, multidrug resistance risk factors have been less studied due to the rarity of this profile. Prior quinolone use, length of stay, and Charlson Comorbidity Index have been linked with isolated TMP–SMX or levofloxacin resistance [1]. However, our results suggest that previous TMP–SMX was strongly associated with the DTR profile, a concerning finding due to its common prophylactic use in immunosuppressed patients. This poses a particular challenge due to the limited therapeutic alternatives available. Among limited alternatives, CZA–ATM had good in vitro activity with an MIC_50/90_ of 1/2 mg/L. Similar results were shown in a recent systematic review (MIC_50/90_ 2/8 mg/L) and the most recent SENTRY Registry (MIC_50/90_ 4/4 mg/L) [8, 18]. Clonal assessment revealed multiple pulsotypes with no dominant clone, consistent with reports from Greece, China, and Peru [19–21]. Considering incidence–carbapenem consumption correlation, these findings suggest selective pressure due to antibiotic prescription as the primary mechanism of emergence, with a minor contribution of hand-to-hand transmission.

Appropriate treatment was strongly associated with lower mortality, as nearly 15% of infections received inappropriate therapy, and most of them died. They received carbapenems, aminoglycosides, or ceftazidime (the latter considered appropriate at the time). These results align with existing literature [22, 23]. Such decisions are likely explained by the need to cover other common agents of ventilator-associated pneumonia susceptible to carbapenems, such as *P. aeruginosa.* A limited of understanding of the intrinsic resistance mechanisms of *S. maltophilia* may be one factor that could have contributed to the absence of appropriate therapy. Therefore, raising awareness among ICU teams by antimicrobial stewardship programmes is essential.

Mortality did not differ between appropriate treatment options, although levofloxacin-containing regimens showed a numerical advantage, consistent with previous reports [24, 25]. Levofloxacin resistance and DTR were associated with worse DOOR outcomes, probably explained by the inability to use levofloxacin. Current guidance suggests combination therapy due to poor in vitro activity of individual agents. However, observational studies report heterogeneous results, ranging from no apparent benefit to a potential advantage in selected subgroups, particularly among patients with severe infections or immunocompromised status [5, 26]. In our study, we did not observe a significant difference between monotherapy and combination therapy. This finding should be interpreted with caution given the retrospective design and limited sample size. Follow-up cultures are not standard practice and were likely obtained due to partial clinical response or concerns regarding treatment efficacy. Nevertheless, it is worth highlighting the high rate of microbiological eradication observed among patients with bacteraemia.

Our study has several limitations. First, selection bias secondary to the retrospective single-centre design, with the possibility of infection misclassification due to the capacity of *S. maltophilia* to colonize the respiratory tract without developing disease. Second, residual confounding cannot be excluded despite multivariable adjustment. Additionally, the use of odds ratios may overestimate effect sizes for more prevalent outcomes; however, most outcomes in our study were of low prevalence. Furthermore, the CZA–ATM synergy test was performed by overlaid strip test as no gold standard had been established. Future studies should include multicentre, prospective cohorts with a larger sample size and with explicit criteria to differentiate colonization from infection. However, our study also has strengths, including a large sample size considering the single-centre nature of the study and a comprehensive approach covering epidemiological, clinical, and microbiological aspects. It is one of the first Latin-American *S. maltophilia* clinical cohorts, reporting an emerging situation of DTR and presents CZA–ATM as an appealing new treatment alternative. Additionally, outcome analysis by DOOR provides a comprehensive framework for assessing patient-centred outcomes.

## Conclusions

*S. maltophilia* has emerged as a relevant carbapenem-resistant gram-negative bacteria in our ICU, with a notably high frequency of DTR phenotypes strongly associated with previous TMP–SMX exposure. Appropriate therapy and levofloxacin susceptibility were linked to better DOOR outcomes, emphasizing the need to understand its intrinsic and acquired resistance mechanisms when selecting treatment. As resistance to first-line agents is rising, ceftazidime/avibactam plus aztreonam is promising due to its in vitro activity and should be further studied in prospective clinical trials.

## Supporting information

10.1017/S0950268826101708.sm001 Ross et al. supplementary material Ross et al. supplementary material

## Data Availability

The data that support the findings of this study are available from the corresponding author, P.R., upon reasonable request.

## Supplementary material

The supplementary material for this article can be found at http://doi.org/10.1017/S0950268826101708.

## Long descriptions

### Table 1. Long description

Table 1 presents clinical and demographic characteristics of 101 patients stratified by four antimicrobial resistance patterns. Rows include: age (median years), sex, obesity, prior hospitalization, Charlson Comorbidity Index (CCI), COVID-19 admission, immunosuppression, surgery during admission, prior S. maltophilia colonization, ICU admission, prior antibiotic use (overall, and by specific agent: TMP-SMX, levofloxacin, ceftriaxone, piperacillin/tazobactam, carbapenems), central venous catheter, invasive mechanical ventilation, renal replacement therapy, SOFA score, and APACHE II score. Statistically significant differences (p<0.05) were observed for CCI (p=0.023), immunosuppression (p=0.017), prior S. maltophilia colonization (p=0.023), prior antibiotic use (p=0.003), and prior TMP-SMX use (p<0.001). DTR patients had notably higher rates of immunosuppression (57.1%), prior colonization (28.6%), and prior TMP-SMX use (71.4%).

Navigate back to Table 1..

### Figure 1. Long description

Panel A is a dual-axis chart from 2017 to 2023. The left Y-axis measures Incidence (cases per 1,000 patients-day) from 0.0 to 3.0. The right Y-axis measures Carbapenem consumption (D D D per 100 patients-day) from 0 to 25. Yellow bars represent consumption, showing a peak in 2021 at approximately 22 units. An orange line represents the incidence rate, showing a steady increase from 0.4 in 2017 to a peak of 2.7 in 2022, before dropping to 1.7 in 2023. A correlation coefficient r equals 0.90 with p equals 0.046 is noted.

Panel B is a stacked bar chart showing resistance percentages for 2021 (N equals 44), 2022 (N equals 35), 2023 (N equals 22), and a Total (N equals 101). The Y-axis ranges from 0% to 100%. Each bar is composed of four categories from bottom to top:

- D T R (pink): 9.1% in 2021, 20% in 2022, 13.6% in 2023, and 13.9% Total.

- S T M P-S M X + R L E V (green): 4.5% in 2021, 8.6% in 2022, 4.5% in 2023, and 5.9% Total.

- R T M P-S M X + S L E V (orange): 9.1% in 2021, 11.4% in 2022, 18.2% in 2023, and 11.9% Total.

- S T M P-S M X + S L E V (blue): 77.3% in 2021, 60% in 2022, 63.6% in 2023, and 68.3% Total.

Navigate back to Figure 1..

### Table 2. Long description

The table contains seven columns: Characteristics, Non-D T R S T M A (n = 87), D T R S T M A (n = 14), p-value, a O R, C I 95%, and a second p-value for the multivariable model.

* Charlson comorbidity index: Non-D T R median 3 (2 to 6); D T R median 4 (3 to 7); univariate p-value 0.091; a O R 1.44; C I 1.04 to 2.00; multivariable p-value 0.025.

* Previous T M P dash S M X use: Non-D T R 8 (9.2%); D T R 10 (71.4%); univariate p-value less than 0.001; a O R 23.15; C I 3.67 to 145.77; multivariable p-value less than 0.001.

* Immunosuppression: Non-D T R 17 (19.5%); D T R 8 (57.1%); univariate p-value 0.002; a O R 2.19; C I 0.33 to 14.45; multivariable p-value 0.414.

* Bacteriaemia: Non-D T R 13 (14.9%); D T R 6 (42.9%); univariate p-value 0.013; a O R 5.51; C I 0.74 to 40.92; multivariable p-value 0.095.

Notes specify that categorical variables are frequency and percentages, while continuous variables are median and interquartile ranges.

Navigate back to Table 2..

### Figure 2. Long description

The figure consists of six panels labeled A through F.

Top Row (Panels A, B, C): Box plots showing Total hospital stay in days on the x-axis (0 to 150) against resistance status on the y-axis. Yellow boxes represent Resistant/D T R and green boxes represent Susceptible/non-D T R. Individual data points are overlaid.

- Panel A: Levofloxacin susceptibility. Resistant (N = 20) and Susceptible (N = 81) both show median stays around 40 to 50 days with similar distributions.

- Panel B: Trimethoprim/sulfamethoxazole susceptibility. Resistant (N = 26) and Susceptible (N = 75) show nearly identical median hospital stays.

- Panel C: D T R phenotype. D T R (N = 14) and non-D T R (N = 87) show consistent median stays near 50 days.

Bottom Row (Panels D, E, F): Stacked bar charts showing D O O R categories by percentage (0 to 100) on the x-axis. The legend for D O O R categories from left to right is: Death (pink), Alive with 3 events (yellow), Alive with 2 events (green), Alive with 1 event (orange), and Alive with no events (blue).

- Panel D: Levofloxacin susceptibility. The Resistant group has a higher death rate (30 percent) compared to the Susceptible group (23.5 percent). The Susceptible group has a larger ‘Alive, no events’ blue segment (16 percent) which is absent in the Resistant group.

- Panel E: Trimethoprim/sulfamethoxazole susceptibility. The Resistant group shows 19.2 percent death and 7.7 percent ‘Alive, no events’. The Susceptible group shows 26.7 percent death and 14.7 percent ‘Alive, no events’.

- Panel F: D T R phenotype. The D T R group has a 21.4 percent death rate and 0 percent ‘Alive, no events’. The non-D T R group has a 25.3 percent death rate and 14.9 percent ‘Alive, no events’.

Navigate back to Figure 2..

### Table 3. Long description

The table is divided into two main sections: A. All patients (n = 101) and B. Only patients with appropriate definitive treatment (n = 87).

Columns from left to right include: Characteristics, Univariable 30-day mortality (No n = 76, Yes n = 25, p-value), and Multivariable 30-day mortality (a O R, 95% C I, p-value).

Section A (All patients) data:

- Age, years: No = 60 (38–68); Yes = 67 (56–76); p = 0.094; a O R = 1.02; C I = 0.98–1.06; p = 0.195.

- Male: No = 50 (65.8%); Yes = 14 (56.0%); p = 0.378; a O R = 0.59; C I = 0.15–2.30; p = 0.453.

- Invasive mechanical ventilation: No = 43 (56.6%); Yes = 20 (80.0%); p = 0.036; a O R = 6.10; C I = 1.07–34.81; p = 0.042.

- A P A C H E II score: No = 15 (9; 19); Yes = 22 (16; 25); p < 0.001; a O R = 1.06; C I = 0.96–1.18; p = 0.195.

- Appropriate definitive treatment: No = 75 (98.7%); Yes = 12 (48.0%); p < 0.001; a O R = 0.01; C I < 0.01–0.17; p = 0.001.

Section B (Appropriate definitive treatment only) data:

- Antibiotic therapy (Levofloxacin): No = 27 (36.0%); Yes = 1 (8.3%); p = 0.057; a O R = 0.12; C I = 0.01–1.11; p = 0.063.

Navigate back to Table 3..

### Figure 3. Long description

Panel A: A dendrogram on the left branches into 21 horizontal rows. Each row corresponds to a P F G E (Pulsed-Field Gel Electrophoresis) lane on the right. The lanes show dark and light gray bands with blue vertical markers indicating specific DNA fragments. The branching structure groups isolates based on the similarity of these band patterns.

Panel B: A donut chart titled Number of isolates per pulsotype. The chart is divided into 11 segments labeled P T 1 through P T 11. Moving clockwise from the top:

* P T 1 (dark blue): 2

* P T 2 (orange): 4

* P T 3 (dark green): 3

* P T 4 (light blue): 2

* P T 5 (magenta): 1

* P T 6 (light green): 4

* P T 7 (navy blue): 1

* P T 8 (brown): 2

* P T 9 (forest green): 1

* P T 10 (teal): 1

* P T 11 (purple): 2

Navigate back to Figure 3..
