# Supplementary material for: Risk factors and outcomes associated with difficult-to-treat resistance in Stenotrophomonas maltophilia: A clinical and microbiological hospital-based cohort study
Source: Epidemiol Infect. 2026 Jun 11;154:e90. doi: 10.1017/S0950268826101708 (PMC13366366; doi:10.1017/S0950268826101708)
Supplement: Ross et al. supplementary material [file S0950268826101708sup001.docx]

**Supplementary material**

Table of Contents

[Figure A1. STROBE flowchart for sample definition 2](#_Toc193365878)

[Figure A2. Gram-negative bacilli incidence in ICU 3](#_Toc193365879)

[Table A1. STROBE Checklist 4](#_Toc193365880)

[Table A2. Desirability of Outcome Ranking (DOOR) criteria used in the present study at 30 days in after index Culture 7](#_Toc193365881)

[Table A3. Multidrug-resistant phenotype risk factors 8](#_Toc193365882)

[Table A4. DOOR characteristics by resistance pattern 9](#_Toc193365883)

# **Figure A1.** STROBE flowchart for sample definition


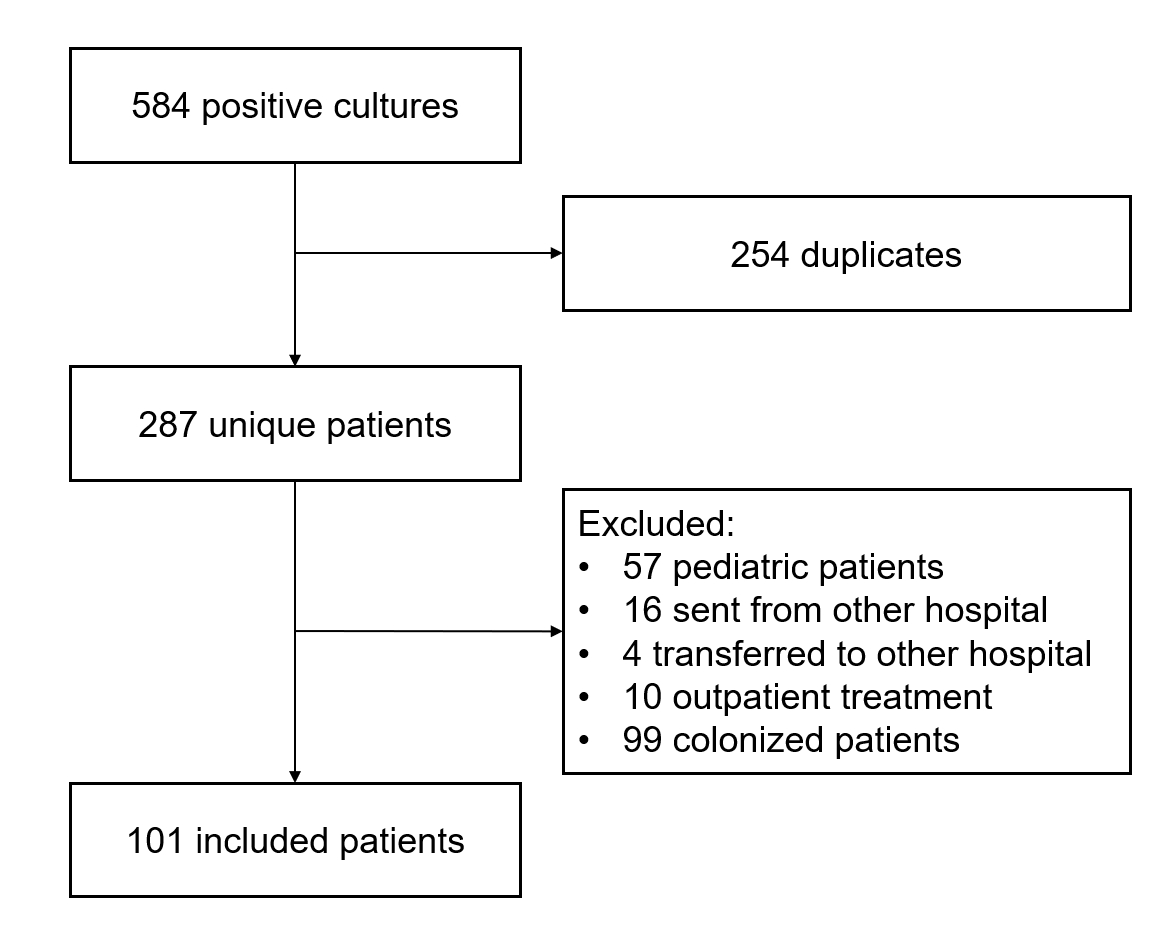


# **Figure A2.** Gram-negative bacilli incidence in ICU

**
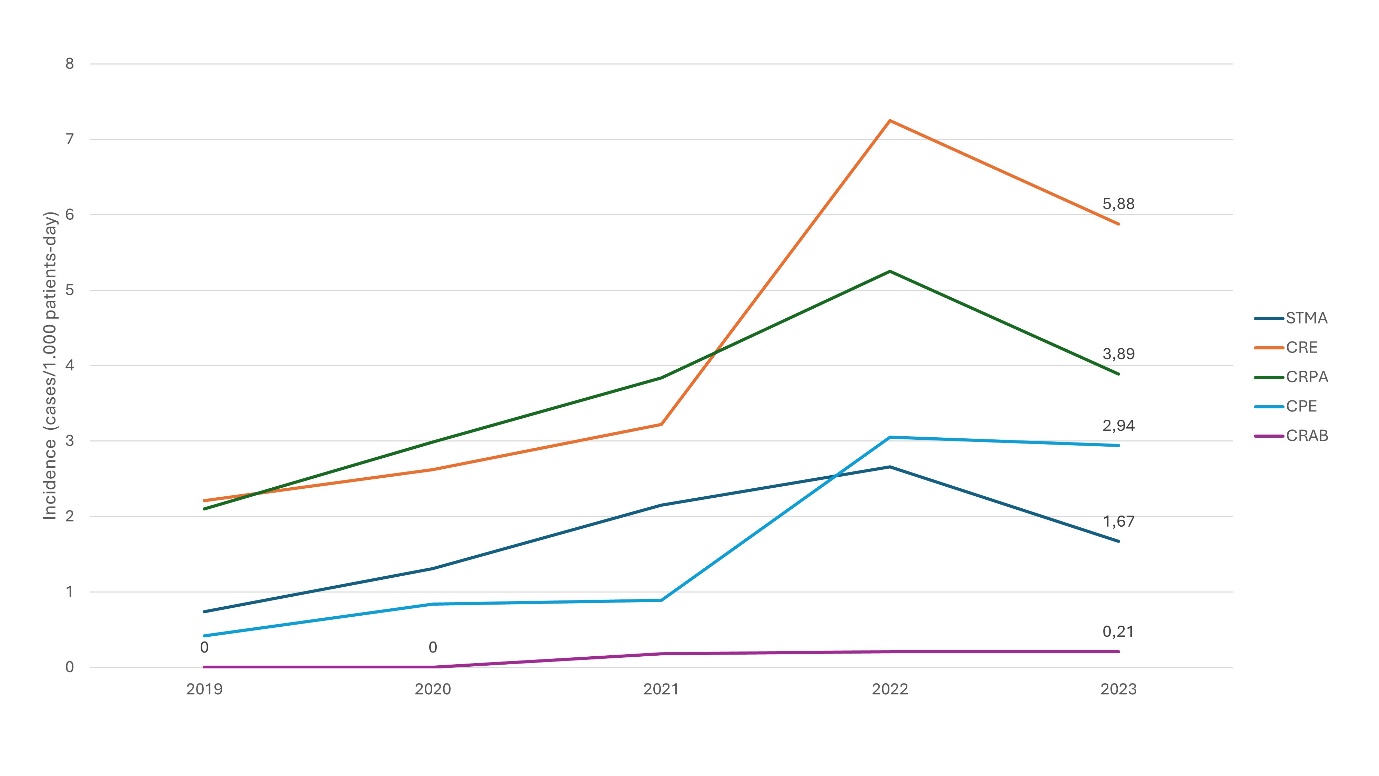
**

STMA: *Stenotrophomonas maltophilia*. CRE: Carbapenem-resistant Enterobacterales. CRPA: Carbapenen-resistante *Pseudomonas aeruginosa.* CPE: Carbapenemase-producing Enterobacterales. CRAB: Carbapenem-resistant *Acinetobacter baumanii/calcoaceticus complex*.

# **Table A1.** STROBE Checklist

|  | Item No | Recommendation | Page |
| --- | --- | --- | --- |
| **Title and abstract** | 1 | (*a*) Indicate the study’s design with a commonly used term in the title or the abstract | 1 |
|  |  | (*b*) Provide in the abstract an informative and balanced summary of what was done and what was found | 2 |
| Introduction | | |  |
| Background/rationale | 2 | Explain the scientific background and rationale for the investigation being reported | 3 |
| Objectives | 3 | State specific objectives, including any prespecified hypotheses | 3 |
| Methods | | |  |
| Study design | 4 | Present key elements of study design early in the paper | 3 |
| Setting | 5 | Describe the setting, locations, and relevant dates, including periods of recruitment, exposure, follow-up, and data collection | 3 |
| Participants | 6 | (*a*) *Cohort study*—Give the eligibility criteria, and the sources and methods of selection of participants. Describe methods of follow-up  *Case-control study*—Give the eligibility criteria, and the sources and methods of case ascertainment and control selection. Give the rationale for the choice of cases and controls  *Cross-sectional study*—Give the eligibility criteria, and the sources and methods of selection of participants | 4 |
|  |  | (*b*) *Cohort study*—For matched studies, give matching criteria and number of exposed and unexposed  *Case-control study*—For matched studies, give matching criteria and the number of controls per case |  |
| Variables | 7 | Clearly define all outcomes, exposures, predictors, potential confounders, and effect modifiers. Give diagnostic criteria, if applicable | 4 |
| Data sources/ measurement | 8* | For each variable of interest, give sources of data and details of methods of assessment (measurement). Describe comparability of assessment methods if there is more than one group | *4* |
| Bias | 9 | Describe any efforts to address potential sources of bias | 3 |
| Study size | 10 | Explain how the study size was arrived at | 3 |
| Quantitative variables | 11 | Explain how quantitative variables were handled in the analyses. If applicable, describe which groupings were chosen and why |  |
| Statistical methods | 12 | (*a*) Describe all statistical methods, including those used to control for confounding | 4 |
|  |  | (*b*) Describe any methods used to examine subgroups and interactions | 4 |
|  |  | (*c*) Explain how missing data were addressed | 5 |
|  |  | (*d*) *Cohort study*—If applicable, explain how loss to follow-up was addressed  *Case-control study*—If applicable, explain how matching of cases and controls was addressed  *Cross-sectional study*—If applicable, describe analytical methods taking account of sampling strategy | -- |
|  |  | (*e*) Describe any sensitivity analyses | -- |

| Results | | |  |
| --- | --- | --- | --- |
| Participants | 13* | (a) Report numbers of individuals at each stage of study—eg numbers potentially eligible, examined for eligibility, confirmed eligible, included in the study, completing follow-up, and analysed | Supp-4 |
|  |  | (b) Give reasons for non-participation at each stage | Supp-4 |
|  |  | (c) Consider use of a flow diagram | Supp-4 |
| Descriptive data | 14* | (a) Give characteristics of study participants (eg demographic, clinical, social) and information on exposures and potential confounders | 6 |
|  |  | (b) Indicate number of participants with missing data for each variable of interest | -- |
|  |  | (c) *Cohort study*—Summarise follow-up time (eg, average and total amount) | -- |
| Outcome data | 15* | *Cohort study*—Report numbers of outcome events or summary measures over time | *6* |
|  |  | *Case-control study—*Report numbers in each exposure category, or summary measures of exposure | *--* |
|  |  | *Cross-sectional study—*Report numbers of outcome events or summary measures | *--* |
| Main results | 16 | (*a*) Give unadjusted estimates and, if applicable, confounder-adjusted estimates and their precision (eg, 95% confidence interval). Make clear which confounders were adjusted for and why they were included | 7 |
|  |  | (*b*) Report category boundaries when continuous variables were categorized | -- |
|  |  | (*c*) If relevant, consider translating estimates of relative risk into absolute risk for a meaningful time period | -- |
| Other analyses | 17 | Report other analyses done—eg analyses of subgroups and interactions, and sensitivity analyses | 7 |
| Discussion | | |  |
| Key results | 18 | Summarise key results with reference to study objectives | 7 |
| Limitations | 19 | Discuss limitations of the study, taking into account sources of potential bias or imprecision. Discuss both direction and magnitude of any potential bias | 8 |
| Interpretation | 20 | Give a cautious overall interpretation of results considering objectives, limitations, multiplicity of analyses, results from similar studies, and other relevant evidence | 8 |
| Generalisability | 21 | Discuss the generalisability (external validity) of the study results | 9 |
| Other information | | |  |
| Funding | 22 | Give the source of funding and the role of the funders for the present study and, if applicable, for the original study on which the present article is based | 9 |

*Give information separately for cases and controls in case-control studies and, if applicable, for exposed and unexposed groups in cohort and cross-sectional studies.

**Note:** An Explanation and Elaboration article discusses each checklist item and gives methodological background and published examples of transparent reporting. The STROBE checklist is best used in conjunction with this article (freely available on the Web sites of PLoS Medicine at http://www.plosmedicine.org/, Annals of Internal Medicine at http://www.annals.org/, and Epidemiology at http://www.epidem.com/). Information on the STROBE Initiative is available at [www.strobe-statement.org.3](http://www.strobe-statement.org.3)

# **Table A2.** Desirability of Outcome Ranking (DOOR) criteria used in the present study at 30 days in after index Culture

| DOOR Score | Criteria |
| --- | --- |
| 0 | Alive, with no events. |
| 1 | Alive, with 1 composite event. |
| 2 | Alive, with 2 composite events. |
| 3 | Alive, with 3 composite events. |
| 4 | Deceased. |

| Event | Definition |
| --- | --- |
| Lack of clinical response | Presence of one or more of the following: lack of symptomatic response, treatment ≥ 30 days, or recurrence. |
| Failed discharge | Presence of discharge ≥ 30 days or rehospitalization. |
| Adverse events | Presence of acute kidney injury (AKI) or Clostridioides difficile infection (CDI). |

Lack of symptomatic response was defined as the absence of improvement in symptoms at discharge if the patient was discharged within 30 days of the index culture. For patients discharged 30 or more days after the index culture, criteria were specific to the site of infection. In cases of bacteremia, a lack of symptomatic response was defined as a failure to achieve a negative blood culture within 7 days. For respiratory infections, it was defined as no improvement in symptoms within 7 days. In urinary tract infections (UTIs), it corresponded to the absence of symptom resolution within 5 days. For intra-abdominal infections, it was defined as the need for continued source control more than 2 days after the index culture. Lastly, in skin and soft tissue infections (SSTIs), a lack of symptomatic response was characterized by less than an 80% reduction in wound size at discharge. Acute kidney injury (AKI) was defined as serum creatinine increase ≥ 3 times the baseline level or initiation of renal replacement therapy (RRT). Patients on RRT prior to the index culture are not eligible.

# **Table A3.** Sample characteristics by year of diagnosis (N=101 patients)

| **Characteristics** | **2021**  **44 (43.6%)** | **2022**  **35 (34.7%)** | **2023**  **22 (21.8%)** | **Total**  **101 (100.0%)** | **T- or χ²-test^☨^ p-value** |
| --- | --- | --- | --- | --- | --- |
| Age (years) | 60 (45; 70) | 63 (43; 73) | 67 (41; 74) | 61 (43; 71) | 0.889 |
| Male sex | 30 (68.2%) | 19 (54.3%) | 15 (68.2%) | 64 (63.4%) | 0.386 |
| **Obesity** | **17 (38.6%)** | **3 (8.6%)** | **3 (13.6%)** | **23 (22.8%)** | **0.003** |
| Previous hospital admission | 8 (18.2%) | 12 (34.3%) | 6 (27.3%) | 26 (25.7%) | 0.262 |
| Charlson Comorbidity Index (CCI) | 3 (2; 6) | 4 (2; 6) | 4 (2; 6) | 3 (2; 6) | 0.863 |
| **COVID-19 as reason for admission** | **26 (59.1%)** | **7 (20.0%)** | **0 (0.0%)** | **33 (32.7%)** | **<0.001** |
| **Immunosuppression** | **6 (13.6%)** | **9 (25.7%)** | **10 (45.5%)** | **25 (24.8%)** | **0.018** |
| Surgery during admission | 10 (22.7%) | 14 (40.0%) | 10 (45.5%) | 34 (33.7%) | 0.113 |
| *S. maltophilia* colonization | 4 (9.1%) | 3 (8.6%) | 4 (18.2%) | 11 (10.9%) | 0.462 |
| Intensive care unit admission | 40 (90.9%) | 31 (88.6%) | 21 (95.5%) | 92 (91.1%) | 0.673 |
| Previous antibiotic use | 40 (90.9%) | 29 (82.9%) | 19 (86.4%) | 88 (87.1%) | 0.565 |
| Ceftriaxone | 21 (47.7%) | 9 (25.7%) | 9 (40.9%) | 39 (38.6%) | 0.132 |
| Piperacillin/Tazobactam | 25 (56.8%) | 17 (48.6%) | 10 (45.5%) | 52 (51.5%) | 0.625 |
| Carbapenems | 16 (36.4%) | 18 (51.4%) | 12 (54.5%) | 46 (45.5%) | 0.259 |
| **Central venous catheter** | **36 (81.8%)** | **18 (51.4%)** | **10 (45.5%)** | **64 (63.4%)** | **0.003** |
| Invasive mechanical ventilation | 31 (70.5%) | 19 (54.3%) | 13 (59.1%) | 63 (62.4%) | 0.316 |
| Renal replacement therapy | 8 (18.2%) | 4 (11.4%) | 5 (22.7%) | 17 (16.8%) | 0.513 |
| SOFA Score | 6 (4; 9) | 4 (1; 8) | 6 (3; 10) | 5 (3; 9) | 0.451 |
| APACHE II Score | 17 (13; 22) | 16 (9; 20) | 16 (10; 23) | 16 (12; 22) | 0.527 |

Notes: Categorical variables expressed as frequency and percentages. Continuous variables expressed as median and interquartile ranges. ☨ T- or χ²-tests were performed following variable’s distribution via comparing mean differences across all sampled years.

# **Table A4.** DOOR characteristics by resistance pattern

|  | MS  69 (68.3%) | R TMP-SMX  12  (11.9%) | R LVX  6  (5.9%) | DTR  14  (13.9%) | Total  101  (100%) | p-value |
| --- | --- | --- | --- | --- | --- | --- |
| DOOR at 30 days |  |  |  |  |  |  |
| alive without events | 11 (15.9%) | 2 (16.7%) | 0 (0.0%) | 0 (0.0%) | 13 (12.9%) | 0.723 |
| alive with 1 event | 17 (24.6%) | 4 (33.3%) | 1 (16.7%) | 6 (42.9%) | 28 (27.7%) |  |
| alive with 2 events | 18 (26.1%) | 4 (33.3%) | 2 (33.3%) | 4 (28.6%) | 28 (27.7%) |  |
| alive with 3 events | 6 (8.7%) | 0 (0.0%) | 0 (0.0%) | 1 (7.1%) | 7 (6.9%) |  |
| dead | 17 (24.6%) | 2 (16.7%) | 3 (50.0%) | 3 (21.4%) | 25 (24.8%) |  |
| DOOR components at 30 days |  |  |  |  |  |  |
| lack of clinical response | 39 (56.5%) | 6 (50.0%) | 5 (83.3%) | 11 (78.6%) | 61 (60.4%) | 0.238 |
| remains on tratment | 2 (2.9%) | 1 (8.3%) | 1 (16.7%) | 1 (7.1%) | 5 (5.0%) | 0.423 |
| recurrence | 8 (11.6%) | 2 (16.7%) | 0 (0.0%) | 3 (21.4%) | 13 (12.9%) | 0.561 |
| not dischardes | 30 (43.5%) | 7 (58.3%) | 3 (50.0%) | 7 (50.0%) | 47 (46.5%) | 0.795 |
| readmitted | 5 (10.0%) | 0 (0.0%) | 0 (0.0%) | 1 (9.1%) | 6 (8.2%) | 0.730 |
| acute kidney injury | 16 (23.2%) | 2 (16.7%) | 0 (0.0%) | 3 (21.4%) | 21 (20.8%) | 0.584 |
| *C. difficile* infection | 3 (4.3%) | 0 (0.0%) | 0 (0.0%) | 0 (0.0%) | 3 (3.0%) | 0.698 |
| 30-day mortality | 16 (23.2%) | 2 (16.7%) | 3 (50.0%) | 3 (21.4%) | 24 (23.8%) | 0.446 |
| 90-day mortality | 21 (30.4%) | 3 (25.0%) | 4 (66.7%) | 6 (42.9%) | 34 (33.7%) | 0.242 |
| Length of stay | 44 (28; 80) | 38 (20; 81) | 42 (24; 72) | 54 (18; 84) | 45 (25; 80) | 0.529 |
| 90-day readmission | 7 (14.0%) | 1 (10.0%) | 0 (0.0%) | 2 (18.2%) | 10 (13.7%) | 0.891 |

MS: Multisusceptible. DTR: Difficul-to-treat resistance
